# Case report: Neuroendocrine carcinoma of the nasal cavity in a roe deer (*Capreolus capreolus*)

**DOI:** 10.3389/fvets.2025.1535432

**Published:** 2025-02-05

**Authors:** Gorazd Vengušt, Diana Žele Vengušt, Carlo Cantile, Mitja Gombač, Kristina Tekavec, Tanja Švara

**Affiliations:** ^1^Institute of Pathology, Wild Animals, Fish and Bees, Veterinary Faculty, University of Ljubljana, Ljubljana, Slovenia; ^2^Department of Veterinary Sciences, University of Pisa, Pisa, Italy

**Keywords:** roe deer, nasal cavity, neuroendocrine carcinoma, tumor, immunohistochemistry

## Abstract

Neuroendocrine tumors of the nasal cavity are rare in both animals and humans. This report describes the macroscopic, histopathological and immunohistochemical characteristics of a neuroendocrine tumor in a three-year-old female roe deer (*Capreolus capreolus*) that was shot due to a facial deformity caused by an oval, firm, exophytic lesion effacing the left frontal and parietal regions. Longitudinal sectioning of the skull revealed a nasal cavity tumor that had invaded the cribriform plate, the rostral bones of the skull, the rostral aspect of the cranial cavity and the frontal sinuses and extended through the lacrimal, sphenoid and zygomatic bones into the subcutaneous tissue. Histopathologically, the tumor consisted of neoplastic cells forming sheets, nests, trabecular and cribriform structures separated by a delicate fibrovascular stroma. Mitoses were rare. Based on the histopathological and immunohistochemical findings, a neuroendocrine carcinoma was diagnosed. Based on thorough database searches, this is the first known case of a nasal neuroendocrine carcinoma in a roe deer.

## 1 Introduction

Tumors in the nasal cavity or paranasal sinuses (i.e., sinonasal tumors) are uncommon in animals. Among the epithelial tumors, adenocarcinomas predominate, whereas chondrosarcoma is the most common malignant mesenchymal tumor (1). Sinonasal tumors with neuroendocrine differentiation, such as neuroendocrine carcinomas (NECs) and olfactory neuroblastomas (ONBs) (formerly known as esthesioneuroblastoma) have been occasionally reported in animals. Namely, NECs have been reported in dogs (*Canis lupus familiaris*) (2), horses (*Equus ferus caballus*) (3) and free-living Japanese raccoon dog (*Nyctereutes procyonoides viverrinus*) (4), whereas ONBs have been described in cats (*Felis catus*) and dogs (2, 5–10), horse (11), cattle (*Bos taurus*) (12), axolotl (*Ambystoma mexicanum*) (13, 14) and goldfish (*Carassius auratus*) (15).

During passive disease surveillance of roe deer in Switzerland (16), Slovenia (17), Sweden (18) and in the Netherlands (19), tumors were detected in 32, 19, 19 and four cases, respectively. The most frequently diagnosed tumor in Slovenia (17) was a fibropapilloma, in Switzerland and Sweden a lymphoma (16, 18), while reports from other researchers suggest that liver neoplasms are the most common tumors in roe deer (20, 21). Other neoplasms of various origins have been described in several case reports, such as pulmonary adenocarcinoma (22), mandibular ossifying fibroma and oral papillomas (23), leukemia (24), neuroblastoma (25), oral squamous cell carcinoma (26), cutaneous teratoma (27), suggesting that this species is particularly prone to developing neoplastic diseases (16).

This report describes the macroscopic, histopathological and immunohistochemical findings of an NEC in a three-year-old female roe deer (*Capreolus capreolus*).

## 2 Case description

**Figure 1:**
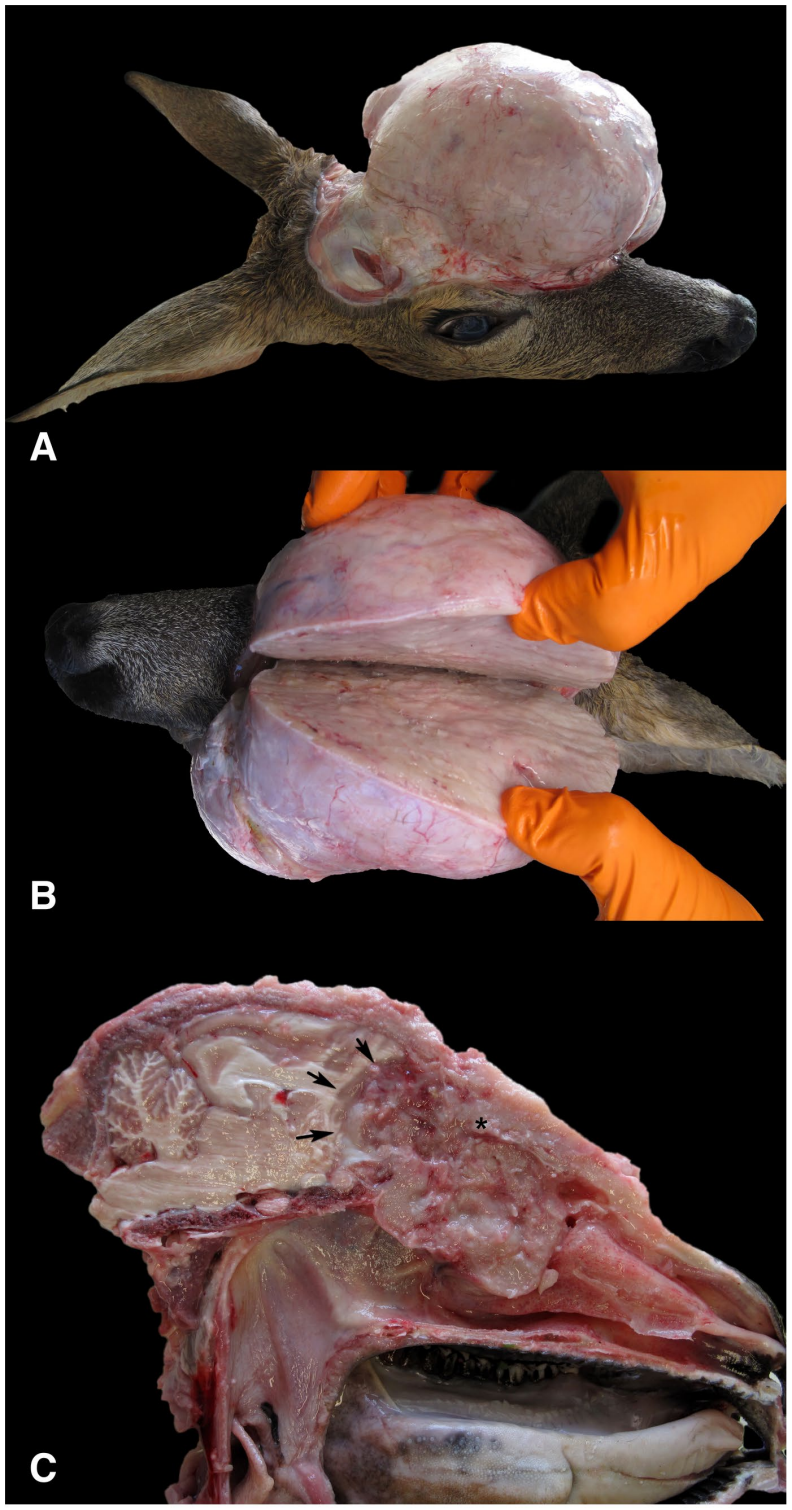
Gross findings in a female roe deer (*Capreolus capreolus*) with neuroendocrine carcinoma. **(A)** The surface of the tumor mass after removal of the skin showed a highly exophytic, grey-white, firm mass. **(B)** The cut surface of the tumor mass was grey-white, smooth and moderately bulged. **(C)** The tumor, which originated from the ethmoidal region of the left nasal cavity (*), had invaded and destroyed the cribriform plate and the rostral bones of the skull. The tumor occupied the rostral part of the cranial cavity and affected the frontal lobe of the brain (arrows). The exophytic portion of the tumor, which protruded above the parietal and nasal regions, was removed.

A three-year-old female roe deer was shot by a local hunter in June 2023 in Nova vas in the Inner Carniola hunting area in southern Slovenia, Europe (45°45′42.89 “N, 14°30′11.43 “E), due to a facial deformity. The age of the animal was estimated by hunter and authorized hunting committees during the mandatory annual inspection of hunted ungulates at the end of the year. Eruption patterns and tooth wear were used to estimate the age of the animal. The animal was submitted to the Veterinary Faculty of the University of Ljubljana for necropsy. At necropsy, the animal was in good body condition (19.5 kg), defined by normally developed skeletal muscles and adequate subcutaneous and internal fat reserves. Gross examination of the head revealed an oval, apparently well-circumscribed, firm lesion in the left frontal and parietal region of the head (Figures 1A,B), which was covered with intact skin. Examination of the cut surface of the head revealed a poorly circumscribed, unencapsulated, oval, grey-white tumor mass measuring 14 × 11.5 × 13 cm, which originated from the ethmoid region of the left nasal cavity and had invaded and destroyed the cribriform plate and the rostral bones of the skull. The tumor occupied the rostral part of the cranial cavity and affected the frontal lobe of the brain. The tumor mass also occupied the frontal sinuses, invaded the frontal, lacrimal, sphenoid and zygomatic bones and grew into the subcutis of the frontal and parietal regions (Figure 1). As a result of the ingrowth of the tumor mass into the orbit, proptosis of the left eye occurred. No metastases and no other lesions were detected at necropsy.

**Figure 2:**
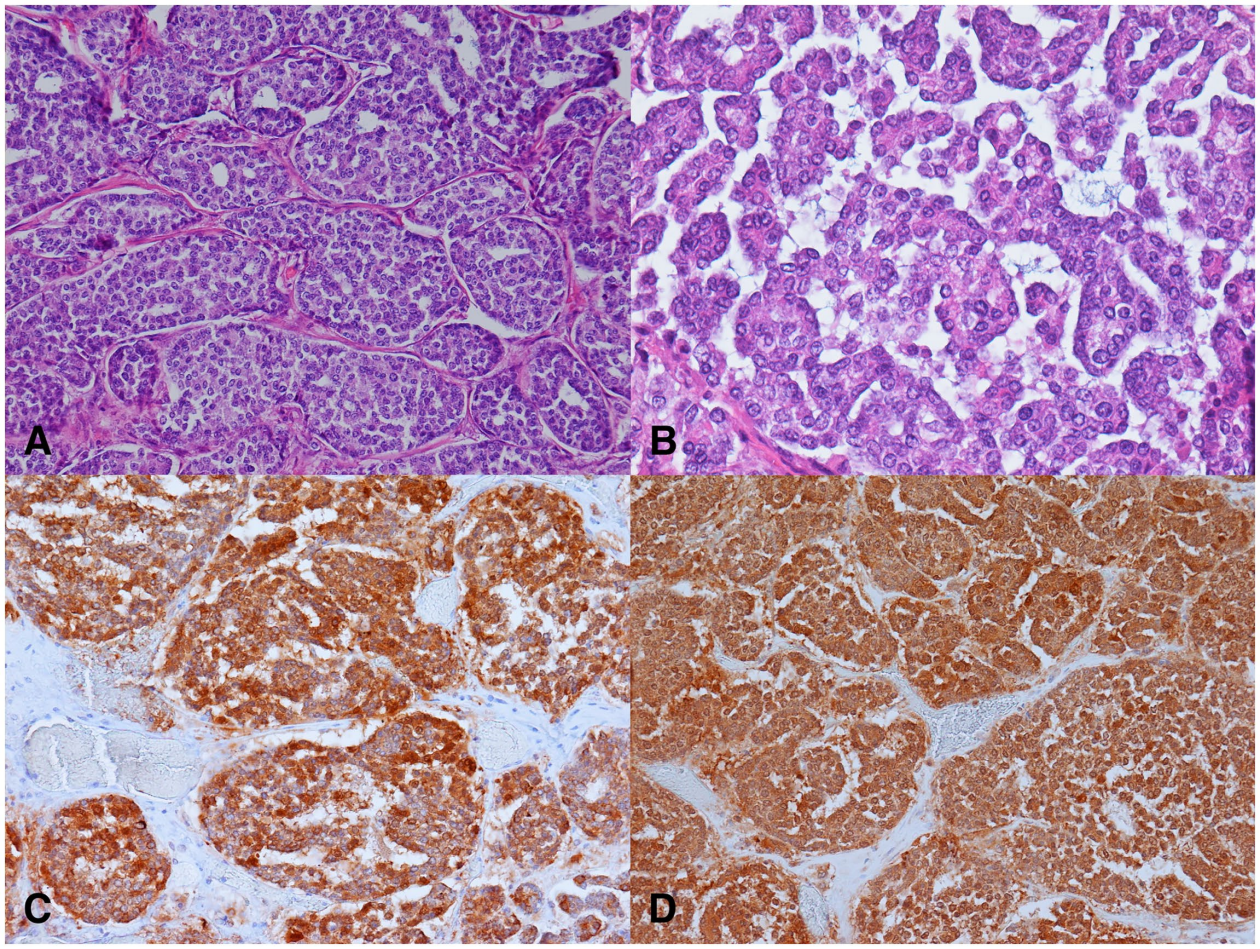
Microscopic findings of the neuroendocrine carcinoma in a female roe deer (*Capreolus capreolus*). **(A)** Nests of neoplastic cells separated by delicate fibrovascular stroma. H&E, 200x. **(B)** Nests of small to medium sized neoplastic cells exhibiting mild anisocytosis and anisokaryosis. H&E, 400x. **(C)** Neoplastic cells demonstrated strong cytoplasmic immunolabeling for cytokeratins. Pancytokeratin AE1/AE3 immunohistochemistry (IHC), 200x. **(D)** Neoplastic cells exhibited strong cytoplasmic immunolabeling for neuron specific enolase (IHC), 200x.

For histopathology, several samples of the tumor were collected, immediately fixed in 10% buffered formalin and routinely embedded in paraffin. For light microscopic examination, 4 μm thick sections were stained with hematoxylin and eosin and with the modified Grimelius stain (28). Histopathological examination revealed predominantly a well-demarcated, partially encapsulated tumor that was multifocally infiltrative and was composed of sheets, nests, trabeculae, rosettes, and cribriform structures separated by a small to moderate amount of fibrovascular stroma. The neoplastic cells were polygonal, with indistinct cell borders and small to moderate amounts of eosinophilic non-granular cytoplasm demonstrating mild anisocytosis. The modified Grimelius staining revealed numerous dark cytoplasmic granules within the neoplastic cells. Single round to oval nuclei were evident, exhibiting mild anisokaryosis and up to one small nucleolus (Figures 2A,B). Three mitoses per 10 high-power fields (2.37 mm^2^) were counted. Multifocally, small to medium sized necrotic foci, hemorrhages and lytic bone fragments were present within the neoplasm. Invasion of blood and lymph vessels was not observed.

Immunohistochemistry was performed to confirm the origin of the neoplastic cells. The procedure was performed on selected 4 μm thick paraffin sections, which were first deparaffinized. The antigens were retrieved by boiling the slides in EDTA buffer (pH 9.0) in a microwave oven for 10 min [for immunolabeling of neuron-specific enolase (NSE), chromogranin A, synaptophysin, and microtubule-associated protein 2 (MAP-2)] or in citrate buffer for 10 min [for immunolabeling of neurofilament H (NFH)] or 20 min [for immunolabeling of cytokeratins, S-100 protein, glial fibrillary acidic protein (GFAP) and calcitonin]. The slides were then incubated with the primary antibodies for 1 h at room temperature in a humidified chamber (Table 1). The remaining immunohistochemistry was performed according to a previously described protocol (29). Tissues from the roe deer without lesions were used as positive controls: pancreas served as a positive control for immunoreactivity for cytokeratins, synaptophysin, chromogranin A, and NSE; thyroid gland served as a positive control for calcitonin; and brain tissue was used for S-100 protein, GFAP, NFH and MAP-2.

**Table 1:** Details of immunohistochemistry reagents and results of immunolabeling.

Antibody	Clonality	Concentration	Incubation	Manufacturer	Immunolabeling in neoplastic cells
Cytokeratin	AE1/AE3	1:100	60 min	DAKO	100%
Cytokeratin	MNF116	1:100	60 min	DAKO	–
Neuron specific enolase		1:100	60 min	DAKO	100%
Synaptophysin		1:100	60 min	DAKO	–
Chromogranin A		1:500	60 min	DAKO	–
Calcitonin		1:1600	60 min	DAKO	–
S-100 protein		1:800	60 min	DAKO	–
GFAP		1: 2000	60 min	DAKO	–
Neurofilament H		1:200	60 min	Millipore	–
Microtubule-associated protein 2 (MAP-2)	HM-2	1:500	60 min	Sigma	–

Most of the neoplastic cells were immunoreactive for cytokeratin AE1/AE3, and NSE. The immunolabeling was intense and localized in the cytoplasm (Figures 2C,D). The neoplastic cells were negative for cytokeratin MNF116, synaptophysin, chromogranin A, calcitonin, S-100 protein, GFAP, and NFH.

Based on the gross, histopathological and immunohistochemical features of the tumor, a nasal NEC was diagnosed.

## 3 Discussion

Based on thorough database searches (PubMed Central, Google Scholar, CAB Abstract) covering the years (1950–2024), and using mixed keyword combinations of “neuroendocrine,” “tumor,” “neoplasm,” “neoplasia,” “carcinoma,” “roe deer,” and “*Capreolus capreolus*,” this is the first description of NEC in a roe deer and the second reported case in wild animals. The first case was previously described in a raccoon dog (4).

Histologically, the neuroendocrine differentiation of the tumor was suspected based on the architecture of the tumor and the morphology of the tumor cells, which were consistent with an endocrine tumor (1). This was confirmed by histochemical and immunohistochemical stainings. The tumor cells showed argyrophilic staining and expressed NSE and cytokeratins. The Grimelius stain is a silver stain that shows neuroendocrine granules with specific peptide hormones and biogenic amines. Although it stains almost all neuroendocrine tumors with few exceptions, it is not specific for a single neuroendocrine tumor type (30). Immunohistochemistry is valuable for the confirmation of tumors of neuroendocrine origin in both humans and animals (2, 4, 31, 32). Cytokeratins, synaptophysin, NSE, and chromogranin A are usually expressed in sinonasal tumors with neuroendocrine differentiation (3, 32). In addition, tumor cells can also express somatostatin, vasoactive intestinal polypeptide (VIP), protein gene product 9.5 (PGP 9.5) (2), and S-100 (4).

In this case, two differential diagnoses were considered: NEC and ONB, based on histopathologic and immunohistochemical confirmation of neuroendocrine differentiation in the tumor cells. Their phenotypic characteristics overlap considerably, which makes differentiation and diagnosis difficult (5, 33).

Grossly, ONB often grows more invasively and causes more extensive destruction of adjacent bony structures (34, 35). Extension of the neoplasm into the olfactory bulb and orbital wall causing proptosis has been described in a cattle with ONB (12). Compression of the olfactory bulb and frontal lobe with caudal displacement of both cerebral hemispheres and the cerebellum has been described in a dog with ONB (36), and osteolysis of the cribriform plate and extension of the tumor into the brain was observed in two dogs and one cat with ONB (7). In the axolotl, the tumor replaced part of the maxillary bone tissue (13). On the other hand, there have been some other cases of ONB in dogs, cats, and horses, in which the tumor showed no invasive growth into the bone and/or brain cavity (6, 11, 37).

In a case of NEC in a Japanese raccoon dog, Kubo et al. (4) reported destruction of the maxilla, extension of the tumor to the subcutis leading to swelling of the ridge of the nose, and extrusion of the eyeball. Sako et al. (2) found neoplastic infiltration of the nasal septum and frontal sinus in two dogs, and osteolysis of the maxilla and frontal bone in two of the ten dogs with NEC included in the study (2). Three horses with NEC were also found to have exophthalmos (3). In the case described here, the tumor also invaded the frontal sinuses, frontal bone, lacrimal bone, sphenoid bone and zygomatic bone as well as the rostral part of the cranial cavity and the orbit, resulting in proptosis of the left eye.

Both NEC and ONB form microscopic rosettes (1), and electron microscopic or immunohistochemical examination is often necessary to differentiate between them. In contrast to NEC, ONB has cell extensions that contain microtubules (1, 4). While the WHO classification requires electron microscopy for a definitive diagnosis in humans (37), these ultrastructural features have not yet been clearly demonstrated in animals (2, 5, 38).

The immunohistochemical findings of NEC and ONB also overlap considerably, and some researchers have even suggested that these two tumors are different manifestations of the same entity (11). Cytokeratins, chromogranin, and synaptophysin, as well as several peptides such as calcitonin and VIP, which are more regularly expressed in NEC (1) and the lack of immunolabeling for some cytoskeletal proteins such as neurofilament, class III beta-tubulin isotype and MAP-2 in human NEC are suggested to be the main differences between NEC and ONB (2, 39). In a study, cytokeratin AE1/AE3 was expressed in all 10 cases of NEC in the nasal cavity of dogs (2). Expression of MAP-2 has been shown to be a potentially reliable and sensitive marker for ONB in dogs and cats, as all but one case of ONB in cats were immunoreactive for MAP-2 (5). MAP-2 expression has also been described in case of ONB in horse (11).

NSE expression is used to support the diagnosis of ONB in human pathology and has also been described in cases of ONB in animals (5, 10). However, in animals, NSE is also expressed in NEC (2).

## 4 Conclusion

In conclusion, NEC in a roe deer was diagnosed based on the histopathological and immunohistochemical characteristics of the tumor. The tumor cells expressed NSE and cytokeratin AE1/AE3, but were immunohistochemically negative for synaptophysin, chromogranin A, calcitonin, S-100 protein, GFAP, NFH, and MAP-2, which in our opinion supports the diagnosis of NEC.

## Data Availability

The original contributions presented in the study are included in the article/supplementary material, further inquiries can be directed to the corresponding author.
